# Supplementary material for: FTY720-P, a Biased S1PR Ligand, Increases Mitochondrial Function through STAT3 Activation in Cardiac Cells
Source: Int J Mol Sci. 2023 Apr 17;24(8):7374. doi: 10.3390/ijms24087374 (PMC10139230; doi:10.3390/ijms24087374)
Supplement: Supplementary file 1 [file ijms-24-07374-s001.zip › ijms-2279370-supplementary.pdf]

**Supplementary Figure 1**

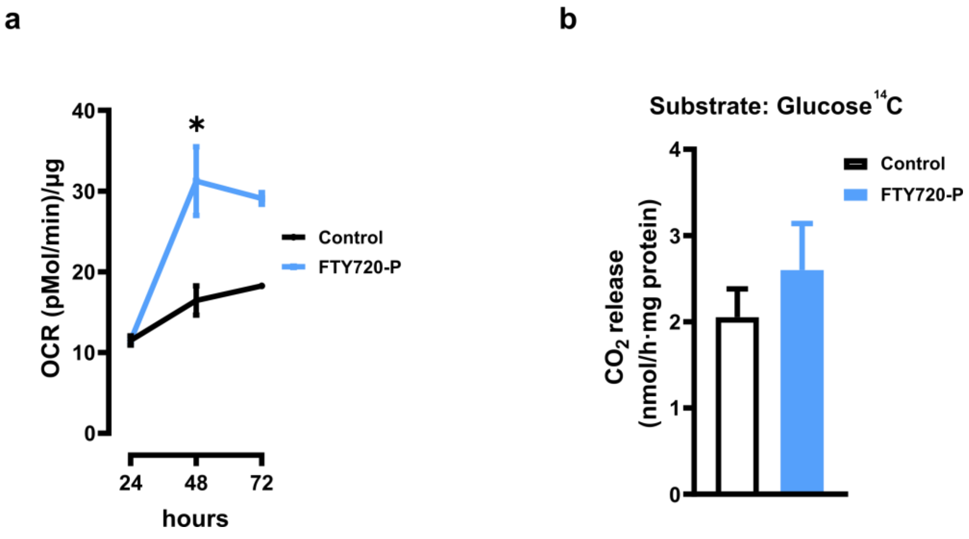


**Supplementary figure S1a)** AC16 cells were incubated with FTY720-P for 24 h, 48 h and 72 h. Thereafter, mitochondrial respiration was analyzed. Data were subjected to unpaired *t*-test. Data are mean ± SEM. * $p$-value < 0.05 vs. control cells. n=3; performed in triplicate.

**Supplementary figure S1b)** AC16 cells were treated with FTY720-P for 48 h and then incubated with (0.1 μCi/mL [U-$^{14}$C] glucose) in KRBH medium for 5 h at 37ºC without $CO_2$ and o/n at room temperature. After, $^{14}CO_2$ precipitated in filter paper soaked with KOH 0.1N were quantified. The detected radioactivity was normalized by total cellular protein.
